# Dexmedetomidine-associated hypothermia in critical trauma: A case report and literature analysis

**DOI:** 10.1097/MD.0000000000041349

**Published:** 2025-01-17

**Authors:** Se Heon Kim, Younghoon Sul, Jin Bong Ye, Jin Young Lee, Jin Suk Lee

**Affiliations:** a Department of Trauma Surgery, Chungbuk National University Hospital, Cheongju, Republic of Korea; b Department of Trauma Surgery, Chungbuk National University College of Medicine, Cheongju, Republic of Korea.

**Keywords:** dexmedetomidine, hypothermia, polytrauma, thermoregulation

## Abstract

**Rationale:**

Hypothermia, defined as a core body temperature below 35°C, is a common and serious complication in severe trauma patients, often worsened by hemorrhage and medical interventions. Dexmedetomidine, an α2-adrenergic agonist used for sedation in intensive care units, has known thermoregulatory effects; however, its association with hypothermia in trauma patients remains insufficiently explored.

**Patient concerns:**

A 40-year-old male with severe polytrauma from a motor vehicle accident presented in distress, with hypotension, tachycardia, and a baseline temperature of 35.8°C. Despite effective management, he developed profound hypothermia, with a recorded temperature dropping below 34.0°C after switching from midazolam to dexmedetomidine for sedation.

**Diagnoses:**

The patient had multiple bilateral rib fractures, a right-sided pneumothorax, and grade 3 liver and grade 5 splenic injuries, along with orthopedic fractures. His Injury Severity Score signified critical trauma, increasing the risk of complications like hypothermia.

**Interventions:**

Following stabilization, dexmedetomidine was administered for sedation. Continuous warming interventions were initiated to address hypothermia; however, the temperature continued to decline. Suspecting dexmedetomidine’s contribution, its administration was discontinued.

**Outcomes:**

After stopping dexmedetomidine, the patient’s temperature gradually recovered to 36.8°C within 5 hours. He demonstrated improved consciousness and stable vital signs, subsequently undergoing 2 successful orthopedic surgeries and discharging without further hypothermia-related issues.

**Lessons:**

This case highlights dexmedetomidine’s potential to induce hypothermia in critically ill trauma patients. It stresses the importance of careful temperature monitoring and proactive thermoregulation during sedative administration in intensive care. Further research is needed to explore the prevalence and mechanisms of dexmedetomidine-associated hypothermia in trauma populations.

## 1. Introduction

Hypothermia is a medical condition characterized by a reduction in the body’s core temperature of < 35°C (95°F), and is relatively common in patients with severe trauma.^[1]^ Hypothermia can result in critically adverse outcomes, particularly in cases of severe trauma characterized by massive hemorrhage. This phenomenon occurs because hypothermia compromises the blood coagulation capacity, thereby intensifying hemorrhage and adversely affecting patient prognosis.^[2]^ The interaction among hypothermia, coagulopathy, and acidosis mutually exacerbates each condition, significantly increasing the mortality rate in patients with severe injuries. This combination, known as the “lethal triad,” is a critical factor that must be addressed when managing patients with trauma.^[1]^ Posttraumatic hypothermia can occur because of hemorrhage, environmental exposure, and medical interventions required for treatment. These medical interventions include massive transfusions, extensive fluid administration, and emergency surgical procedures as well as the administration of medications for sedation and symptomatic relief.^[3]^ Dexmedetomidine, an α2-adrenergic agonist, is frequently used for sedation in intensive care units (ICUs), particularly in patients with severe trauma.^[4]^ Numerous studies have demonstrated the safety and efficacy of dexmedetomidine compared with other sedatives, including its benefits in reducing delirium and shortening ICU stays.^[5,6]^ However, dexmedetomidine has side effects, with hypotension and bradycardia being notably common.^[7]^ In rare instances, they can influence thermoregulation and lead to adverse effects.^[8,9]^ Previous reports have indicated that thermoregulation issues were primarily observed in patients with fever, with 1 documented case of hypothermia induced in a newborn.^[10]^ Herein, we present the case of an adult patient with polytrauma who developed hypothermia after dexmedetomidine administration for sedation, along with a comprehensive review of the related literature.

## 2. Case presentation

**Figure 1.**
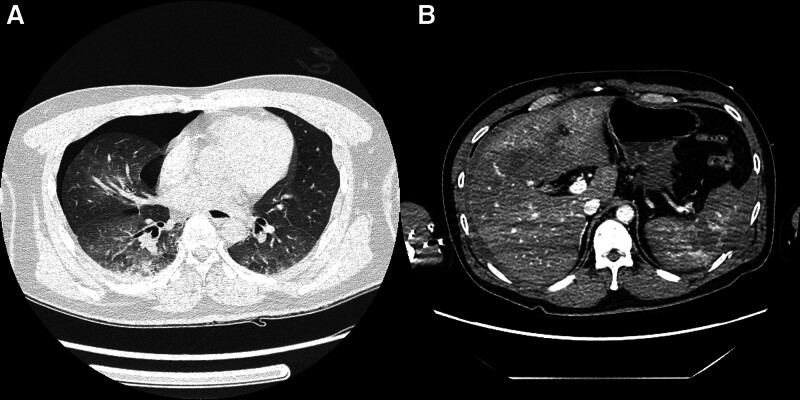
Abnormalities detected on the chest and abdominal computed tomography (CT) of the patient. (A) Posttraumatic chest computed tomography (CT) revealed a right-sided pneumothorax. (B) Grade III liver injury and Grade V splenic injury, accompanied by hemoperitoneum, were observed. CT = computed tomography.

**Figure 2.**
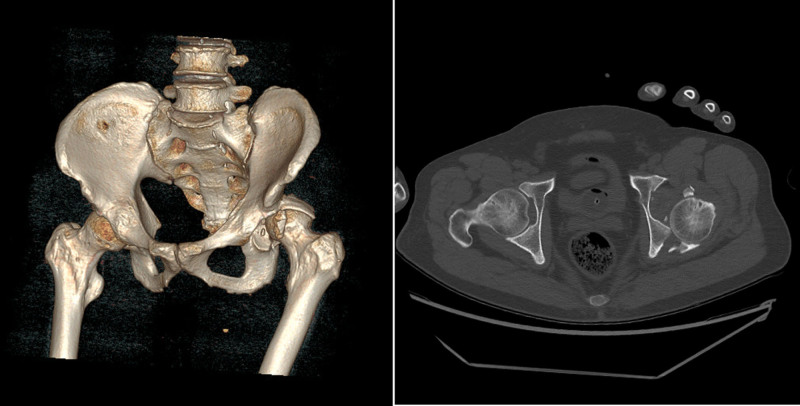
A 3D-reconstructed acetabular fracture visualized by pelvic computed tomography (CT) imaging. CT = computed tomography.

**Figure 3.**
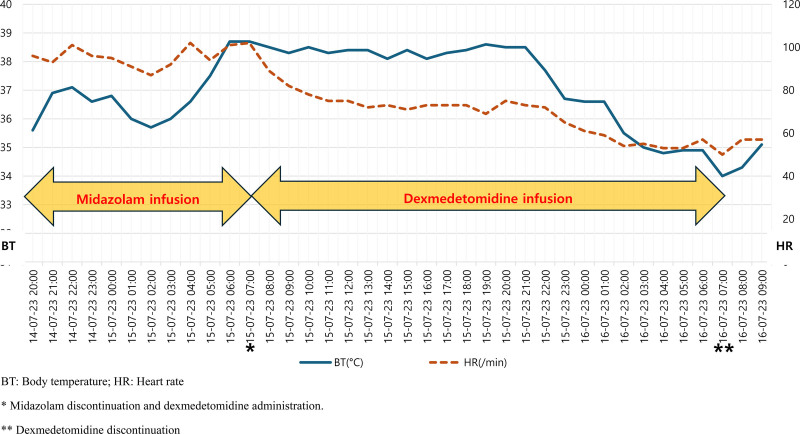
Changes in body temperature and heart rate during the initial 24 hours. *Midazolam discontinuation and dexmedetomidine administration. **Dexmedetomidine discontinuation. BT = body temperature, HR = heart rate.

**Figure 4.**
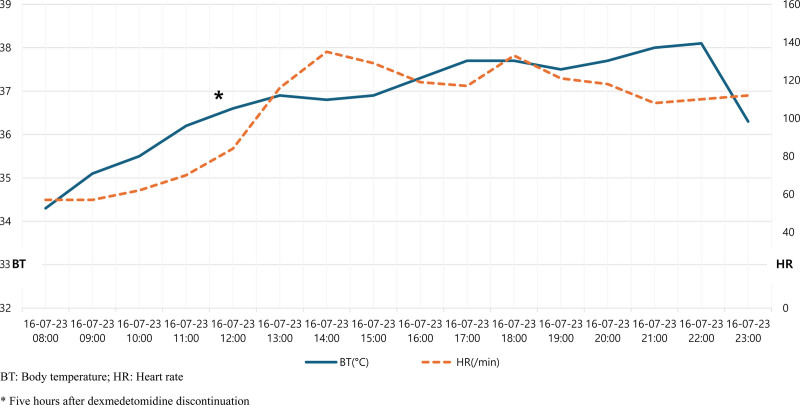
Changes in body temperature and heart rate after dexmedetomidine discontinuation. *Five hours after dexmedetomidine discontinuation. BT = body temperature, HR = heart rate.

A male patient in his late 40s presented to a regional trauma center with severe pain in the right pelvis, knee, and back following a motor vehicle accident. Although the patient experienced a loss of consciousness at the time of the accident, his level of consciousness improved upon hospital admission. The patient presented with dyspnea and significant pain. The initial vital signs recorded were as follows: blood pressure, 80/50 mm Hg; heart rate, 140 beats/minute; respiratory rate, 30 breaths/minute; oxygen saturation, 84%; and body temperature, 35.1°C. The trauma team promptly initiated airway management via intubation and implemented a massive transfusion protocol. Following the initial resuscitation efforts, imaging examinations were performed to evaluate the extent of trauma. The results revealed multiple bilateral rib fractures (right: 5th–8th ribs; left: 5th–10th ribs), right-sided pneumothorax, grade 3 liver injury, and hemoperitoneum associated with grade 5 splenic injury (Fig. 1). Further diagnoses included left hip dislocation with an accompanying acetabular fracture, left distal femoral open fracture, right distal femoral fracture, bilateral patellar fractures, and right foot fracture (Fig. 2). Following emergency medical intervention, the patient underwent angioembolization to manage the hemoperitoneum resulting from liver and spleen injuries. During the angioembolization procedure to control the hemorrhage, the patient received transfusions comprising 8 units of red blood cells and 5 units of fresh frozen plasma. Upon completion of the intervention, the patient’s systolic blood pressure stabilized (90 mm Hg). The patient was subsequently admitted to the trauma intensive care unit for continuous monitoring and further treatment. Upon trauma intensive care unit admission, the patient’s vital signs were as follows: blood pressure, 110/60 mm Hg; heart rate, 80 beats/minute; respiratory rate, 20 breaths/minute; and body temperature, 35.8°C. The medical team opted to continue sedation and ventilatory support to manage shock by initially administering midazolam and remifentanil. On the first day after admission, additional sedative treatment was administered to facilitate postoperative care and recovery. Consequently, the sedation regimen was adjusted by switching between midazolam to dexmedetomidine. Dexmedetomidine was initiated at 0.5 mcg/kg/hour, and remifentanil was concurrently infused at 0.1 mcg/kg/minute. Throughout this period, the patient maintained a Richmond Agitation-Sedation Scale score of −4 while being managed using pressure-controlled ventilation modes. Twelve hours after the initiation of dexmedetomidine, the patient’s body temperature was elevated, fluctuating between 38.0 and 38.7°C. Subsequently, the body temperature began to decrease steadily after this 12-hour period (Fig. 3). Nineteen hours after dexmedetomidine administration, the patient’s body temperature decreased to 35.5°C. Despite the implementation of continuous warming therapy, the body temperature continued to decline, registering <34.0°C approximately 24 hours postadministration (Fig. 3). The patient exhibited decreased heart rate and increased urine output per hour, accompanied by reduced body temperature. Notably, no decrease in the blood was observed during this period. When hypothermia was observed, the patient was administered crystalloid fluids at 120 cc/hour, and sedative agent concentrations were maintained at the previously specified dosages without modification. Laboratory assessments revealed no evidence of acidosis or findings indicative of coagulopathy. Furthermore, physical examination did not reveal any abnormalities, including additional bleeding or other factors likely to contribute to hypothermia as a result of sustained traumatic injuries. The medical team suspected that the symptoms could be attributed to the side effects of dexmedetomidine, which has been used as a sedative in mechanical ventilation and shock management. Consequently, a decision was made to discontinue drug administration. Following the discontinuation of dexmedetomidine, the patient’s body temperature gradually returned to normal, reaching 36.8°C approximately 5 hours after discontinuation (Fig. 4). Subsequently, the patient exhibited progressive recovery of consciousness, vital signs remained stable, and administration of sedation and other pharmacological agents to maintain blood pressure was terminated. During hospitalization, the patient underwent 2 orthopedic surgeries under general anesthesia and demonstrated satisfactory recovery. Notably, no further instances of hypothermia were observed throughout the hospital stay, and the patient was discharged.

## 3. Discussion

### 3.1. Hypothermia in severe trauma

Hypothermia is a common condition in patients with severe trauma and is exacerbated in conjunction with traumatic hemorrhage or hypovolemic shock.^[1]^ Post-traumatic hypothermia immediately after trauma can be considered accidental hypothermia.^[3]^ Factors such as low ambient temperature, wet clothing at the time of injury, administration of cold fluids during resuscitation, and high injury severity are recognized as significant risk factors contributing to hypothermia in trauma patients.^[3,11,12]^

### 3.2. Trauma severitiy and management overview

In the present case, the patient sustained severe trauma (Injury Severity Score: 43). This highly severe of injury necessitated a massive transfusion protocol comprising 8 units of red blood cells and 5 units of fresh frozen plasma, as well as mechanical ventilation, including intubation. These factors significantly increased the risk of hypothermia. However, this case should not be classified as accidental hypothermia, as the patient’s body temperature returned to normal following active damage control resuscitation and warming interventions, with subsequent recurrence of hypothermia.

### 3.3. Recurrence of hypothermia

This phenomenon can be characterized as a situation in which body temperature declines anew owing to various additional factors, including further hemorrhage, surgical interventions, side effects of medications, and environmental conditions encountered during trauma treatment or hospitalization. Such cases typically arise from a confluence of factors, wherein large-volume transfusions, infections, or physiological alterations resulting from the traumatic event can significantly influence thermoregulation.^[1,11]^

### 3.4. Role of sedatives

Considering the potential side effects of medications administered during treatment, it is plausible that sedatives, such as remifentanil, midazolam, or dexmedetomidine, may have contributed to the recurrence of hypothermia. While remifentanil and midazolam are not typically recognized as a direct inducer of hypothermia, they can contribute to hypothermic conditions by impairing the body’s thermoregulatory mechanisms, including shivering and vasoconstriction.^[13,14]^ However, in this case, hypothermia persisted despite the discontinuation of midazolam. Even when hepatic impairment resulting from trauma is considered, it is challenging to attribute hypothermia to midazolam, particularly considering its elimination half-life.

### 3.5. Impact of dexmedetomidine

Although the potential impact of remifentanil on hypothermia was not assessed in this case, the clinical pattern indicating resolution of hypothermia following discontinuation of dexmedetomidine suggests that dexmedetomidine was the likely causative agent of hypothermia. Several cases of body temperature changes due to dexmedetomidine use have been reported, and unlike this case, most studies have reported increased body temperature in ICU patients.^[8,9,15–17]^ Additionally, long-term dexmedetomidine exposure is correlated with an increased maximum temperature.^[18]^

### 3.6. Mechanisms of hyperthermia

While, there are numerous potential etiologies for hyperthermia induction, including infection, dehydration, malnutrition, and stress. Determinants related to central regulatory centers, such as the hypothalamus, as well as environmental factors, are integral to the mechanisms underlying hyperthermia. Further research is required to elucidate these relationships.^[18]^

### 3.7. Bradycardia and concurrent symptoms

Conversely, although instances of hypothermia are relatively rare, bradycardia associated with hypothermia has been documented in neonates who received dexmedetomidine.^[10]^ Similarly, the patient in this report exhibited concurrent hypothermia and bradycardia, with both symptoms returning to normal after discontinuation of dexmedetomidine.

### 3.8. Effects during anesthesia

Furthermore, hypothermia was noted in a study investigating body temperature variations associated with dexmedetomidine administration under general anesthesia during surgical procedures.^[19]^ Cruz et al^[19]^ reported no significant changes in body temperature during surgical procedures; however, variations in body temperature were noted during the postoperative monitoring period following the discontinuation of anesthetic agents. Hypothermia was documented in 3 of 42 patients during this postoperative observation.

### 3.9. Thermoregulation mechanisms

The decrease in body temperature that occurs during general anesthesia for surgery can be attributed to the suppression of the body’s thermoregulatory mechanisms by anesthetic agents. In contrast, dexmedetomidine administration contributes to heat loss by lowering the thresholds for shivering and vasoconstriction, thereby impairing the body’s ability to maintain normothermia.^[20,21]^

### 3.10. Understanding dexmedetomidine’s impact on thermoregulation

Previous animal experiments have reported that α2-adrenergic agonists can cause severe hypothermia.^[22,23]^ Dexmedetomidine is a representative α2-adrenergic agonist approved by the United States Food and Drug Administration.^[24]^ α2-adrenergic agonists are recognized for their influence body temperature regulation, primarily by modulating the secretion and activity of neurotransmitters associated with thermoregulation in the hypothalamus. Furthermore, these agents induce a decrease in metabolic heat production through activation of α2-adrenergic receptors.^[25]^ Previous studies have indicated that prolonged administration of dexmedetomidine in the ICU can decrease body temperature.^[19,20]^ Therefore, in cases such as the one presented in which hypothermia was observed 24 hours after the administration of dexmedetomidine, it is reasonable to consider dexmedetomidine as a potential contributing factor to the patient’s hypothermia.

## 4. Limitations

This case study presents important findings regarding dexmedetomidine-associated hypothermia; however, it is essential to acknowledge several limitations. Firstly, as a single case report, the generalizability of these findings to broader populations may be limited. Additionally, temperature was assessed using tympanic methods rather than direct core temperature measurements, which may impact accuracy. Other confounding factors such as the simultaneous use of additional sedatives and fluid management strategies were not thoroughly controlled or documented, making it challenging to isolate the role of dexmedetomidine. Variations in individual patient responses to medications also pose a challenge in drawing broad conclusions. Finally, the lack of extensive follow-up data limits insights into potential long-term repercussions of dexmedetomidine on thermoregulation. Future studies involving larger cohorts and standardized monitoring protocols will be crucial to deepen our understanding of this phenomenon in critically ill trauma patients.

## 5. Conclusion

Among critically ill patients, changes in body temperature have several causes, such as high fever or hypothermia. In particular, dexmedetomidine, which is used for sedation, affects the thermoregulatory function of the hypothalamus and the threshold for shivering vasoconstriction. Therefore, active temperature monitoring and medical staff awareness of the potential effects of dexmedetomidine are necessary.

## Acknowledgments

We would like to thank Editage (www.editage.co.kr) for the English language editing.

## Author contributions

**Conceptualization:** Younghoon Sul.

**Data curation:** Se Heon Kim.

**Formal analysis:** Se Heon Kim, Jin Young Lee, Jin Suk Lee.

**Resources:** Jin Bong Ye.

**Writing – original draft:** Se Heon Kim.

**Writing – review & editing:** Se Heon Kim, Younghoon Sul, Jin Bong Ye, Jin Young Lee, Jin Suk Lee.
